# Cone Beam Computed Tomography Evaluation of Stafne Bone Defect: A Case Series and Review of Radiographic Features

**DOI:** 10.1155/crid/4058835

**Published:** 2025-04-03

**Authors:** Ibrahim Yamany, Hanadi Sabban

**Affiliations:** Oral Diagnostic Sciences Department, Faculty of Dentistry, King Abdulaziz University, Jeddah, Makkah Province, Saudi Arabia

## Abstract

**Background:** Stafne's bone defects (SBDs) are rare, intraosseous lesions not only localized in the mandible but also asymptomatic by default and found occasionally at radiographically investigations. The size and location of these defects can vary, although most are located in the posterior mandible. Since anterior variants are less frequently reported, diagnostic imaging is crucial for distinguishing SBDs from other diseases. This case series documents both familiar and unusual appearances, highlighting the diagnostic value of cone beam computed tomography (CBCT) in the evaluation of SBDs.

**Case Presentation:** This study evaluated four instances of SBDs using CBCT. In Case 1, a 48-year-old man without any clinical symptoms had a characteristic posterior SBD located beneath the inferior alveolar canal. Case 2 described a 28-year-old woman's unusual anterior mandibular SBD, which was accompanied with scalloping and tooth diversion. In Case 3, a 59-year-old woman had a unique case of bilateral SBDs with combined buccal and lingual depressions. In Case 4, a 47-year-old man had a large anterior SBD with partial root exposure and fenestration. In all cases, CBCT interpreted detailed three-dimensional imaging, assessing diagnosis and effective differentiation from other mandibular pathologies.

**Conclusions:** CBCT proved to be essential in accurately diagnosing SBDs in every instance, particularly when it came to spotting unusual signs like anterior and bilateral lesions. The findings confirmed that SBDs are benign and typically do not require treatment. The improved radiographic capabilities of CBCT support the argument for conservative management strategies and improve diagnostic accuracy.

## 1. Introduction

Stafne's bone defect (SBD), which is known as lingual mandibular bone depression or Stafne's bone cavity/cyst, is a rare and mostly asymptomatic lesion of the mandible [1, 2]. It was described first by Edward C. Stafne in 1942; this condition has since been the subject of many studies and case reports [3, 4]. SBD is typically described radiographically as a well-defined, unilocular radiolucency in the posterior mandible, located below the inferior alveolar canal and close to the angle of the mandible [1, 5]. However, variants have been documented in other locations, including the anterior mandible and mandibular ramus [6, 7]. The reported prevalence of SBD ranges from 0.08% to 0.48%, with a strong male predilection [2, 8]. The most commonly recognized hypothesis of SBD states that the deficiency is caused by pressure from the submandibular salivary gland on the mandibular lingual surface, although the precise cause of the condition is still up for debate [1, 9]. Other suggested causes consist of vascular abnormalities, bone destruction associated with endocrine problems, and growth anomalies occurring during the ossification of the mandible. Previously, SBD has been discovered using conventional radiographic techniques such as panoramic radiography. However, the ability of these two-dimensional imaging modalities to precisely represent the three-dimensional aspects of the defect remains limited [10–12]. Cone beam computed tomography (CBCT) has recently shown potential as a diagnostic tool for SBD [13, 14]. This is due to its three-dimensional visualization, enhanced characterization, and less radiation exposure in comparison with multidetector computed tomography (MDCT). However, radiation dosage is greater than panoramic radiography. Finally, CBCT provides potential predictive capability for assessing the soft tissue content within the lesion [13–16].

CBCT has been shown to be a reliable diagnostic and characterization tool for SBD in several studies. Sisman et al., for instance, showed that CBCT is more effective than panoramic radiography at accurately assessing the features and contents of SBDs. For the purpose of diagnosing an atypical, significant SBD, Li et al. investigated the use of CBCT sialography, highlighting its potential for relating the defect to neighboring salivary gland tissue. Adisen et al. demonstrated the potential of CBCT for quantitative analysis of SBDs through conducting volumetric assessments for such lesions. Furthermore, their study observed a beneficial correlation between the size of the lesion and its distance to the mandibular canal, which could prove clinically significant for surgical planning if intervention is required.

However, it is important to acknowledge that SBD is usually an incidental finding and does not require treatment [1, 4]. In the management of SBD, CBCT is mainly used to confirm the diagnosis and rule out other, possibly more serious diseases that may require clinical intervention [3, 7]. The introduction of CBCT also significantly improved our capacity to identify and describe these lesions, even though SBD is still an uncommon disorder [3, 5, 7]. SBDs can be distinguished from other mandibular pathologies with the assistance of the three-dimensional image analysis of CBCT, which offers beneficial interpretation of their dimensions, shape, and relationship to surrounding structures [5, 7]. The CBCT technology is anticipated to become more significant in the treatment of SBD and other maxillofacial lesions as it develops [3, 5, 7].

## 2. Case Presentation

The following four cases were sourced from the Oral Radiology Department at King Abdulaziz University Dental Hospital, where each patient was interpreted using CBCT. All CBCT scans were obtained using the i-Cat Next Generation machine (Imaging Science International, Hatfield, Pennsylvania) with 120 kV and 37 mAs for the large volume CBCT scans and the KaVo OP 3D Pro machine (KaVo Dental GmbH, Biberach, Germany) with 90 kV and 18.4 mAs for the limited view CBCT. OnDemand3D software (CyberMed, Seoul, South Korea) was used to view the scans, which were diagnosed at the oral and maxillofacial radiology department. The radiographic findings, along with relevant clinical data, are described individually in the following sections.

### 2.1. Case 1

**Figure 1:**
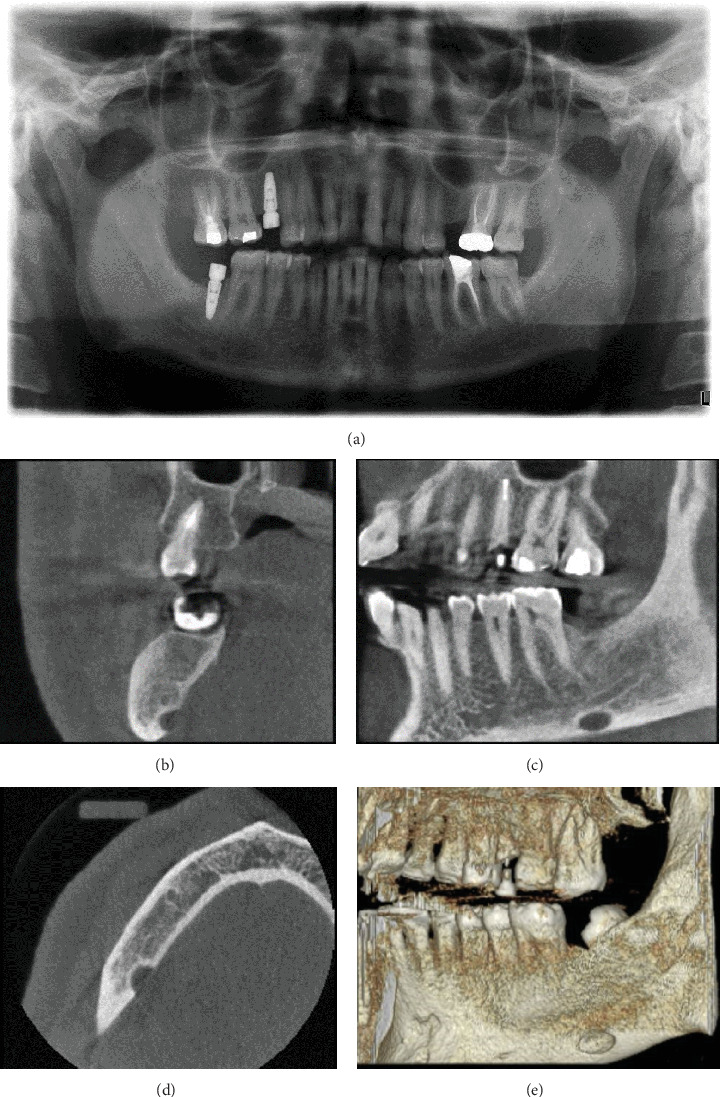
Case 1. (a) Panoramic radiograph of the Stafne bone defect in the right body of the mandible. The image shows a well-defined corticated radiolucent oval shaped entity below the inferior alveolar canal. (b–e) Multiplanar reconstruction (MPR) view of the CBCT scan illustrating (b) corrected coronal, (c) corrected sagittal, (d) axial, and (e) 3D rendering images, representing the lingual position of the defect and its inferior relation to the mandibular canal.

A 48-year-old male patient visited for a routine dental implant procedure and a limited view CBCT scan (260 × 260 mm); the resolution of 0.3 mm voxel size was done. During the radiographic examination for the scan, a SBD was incidentally identified with the following radiographic findings. This lesion was located beneath the inferior alveolar nerve and towards the tongue side, within the mandible, parallel to the location of Tooth #37. It is characterized as well defined, corticated, radiolucent, and oval in shape, measuring 4.8 mm in height, 8.7 mm in buccolingual width, and 3 mm in anteroposterior depth. Notably, the lesion presented a 3-mm depression on the lingual buccal cortex, with no signs of displacement in adjacent structures. Due to its typical radiographic features and the absence of symptoms, no histopathological assessment or treatment was deemed necessary, in accordance with standard protocols for managing common cases of SBD. Follow-up is recommended for these lesions for monitoring lesion stability and early detection of atypical features (Figures 1, 1, 1, 1, and 1).

### 2.2. Case 2

**Figure 2:**
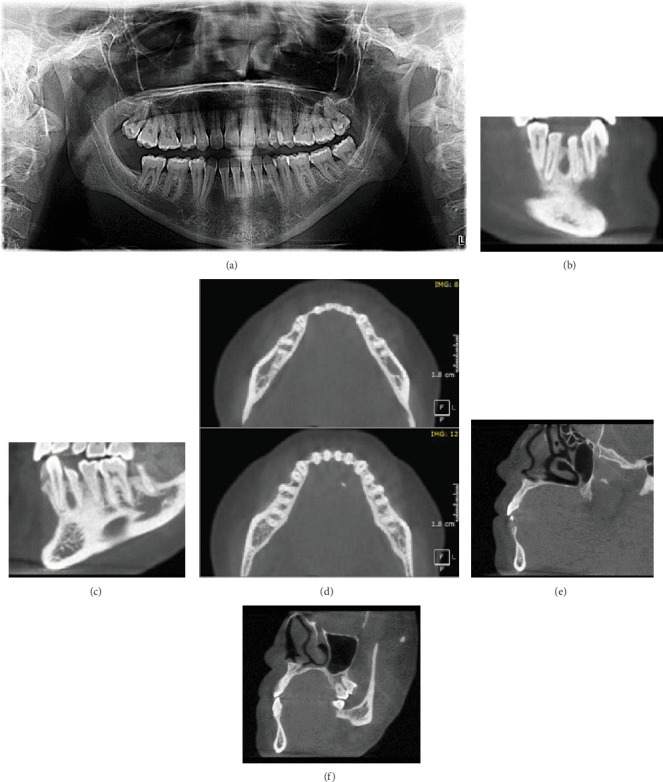
Case 2. (a) The panoramic radiograph shows a radiolucent entity between Teeth #42 and #43 in the anterior mandible. The canine exhibits mesial dilaceration. (b–f) CBCT scan with MPR views: (b, c) corrected sagittal, (d) two levels of axial sections, and (e, f) corrected coronal. (b, e) A significant lingual bone depression between Teeth #43 and #42. (c, f) The contralateral area between Teeth #32 and #33. (d) The axial sections represent all lingual depressions in the anterior mandible.

A 28-year-old female patient presented for surgical consultation regarding an entity between Teeth #42 and #43. The history of the patient revealed that she previously received an orthodontic treatment. Radiographic examination was done by a large volume CBCT scan (640 × 640 mm); the resolution is 0.25 mm voxel size. The radiographic interpretation for the scan presents a well-defined radiolucent fossa (radiolucency) on the lingual surface of the lower anterior mandible, measuring 10 × 5 × 3.5 mm between Teeth #42 and #43. The lesion caused diversion of the adjacent teeth and exhibited similar depressions in the interdental anterior teeth, radiographically interpreted as scalloping borders. The thinning of the alveolar bone between Teeth #42 and #43 is more significant than the other lingual depressions. This unusual presentation in the anterior mandible, along with its effect on tooth location, challenged the differential diagnosis of a SBD. This case highlights the advantages of taking the history of dental treatment, as it can significantly influence bone alterations associated with issues related to dental alignment (Figures 2a, 2b, 2c, 2d, 2e, and 2f).

### 2.3. Case 3

**Figure 3:**
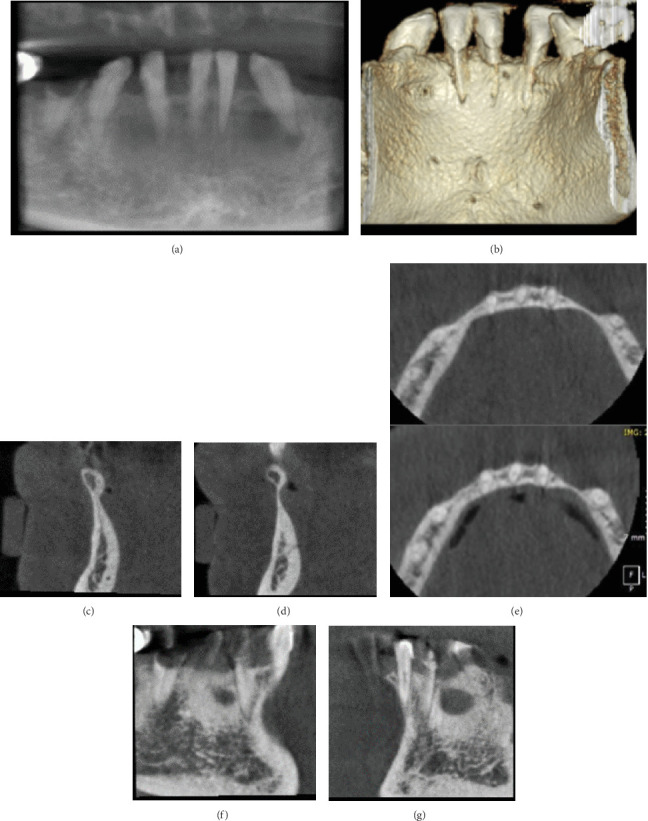
Case 3. CBCT scan. (a) Panoramic reconstruction of CBCT showing bilateral bone radiolucency in the anterior mandible. (b) 3D rendering. MPR views: (c, d) corrected coronal, (f, g) corrected sagittal, and (e) two levels of axial sections. These images show bilateral lingual bone depression for area between Teeth #32 and #33 and area between Teeth #42 and #43. These depressions bilaterally representing the differential diagnosis of Stafne bone defect.

A 59-year-old female patient was referred from a student clinic for surgical extraction of Tooth #32 and further assessment of a radiolucent area located in the anterior mandible. CBCT imaging was performed by limited view scan (250 × 250 mm); the resolution is 0.2 mm voxel size. It revealed the presence of bilateral lingual depressions in the lower anterior mandible, with dimensions of 4 × 6.4 mm on the left side and 3.5 × 4.8 mm on the right side. Notably, there was evidence of both buccal and lingual bone loss, leading to diversion of adjacent teeth due to these radiolucent depressions. This case illustrates an uncommon occurrence of mandibular bone defects characterized by bilateral presentation and simultaneous buccal and lingual involvement in the anterior region. The distinctive aspects of this case highlight the critical role that advanced imaging techniques play in diagnosing and managing suspected SBDs (Figures 3a, 3b, 3c, 3d, 3e, 3f, and 3g).

### 2.4. Case 4

**Figure 4:**
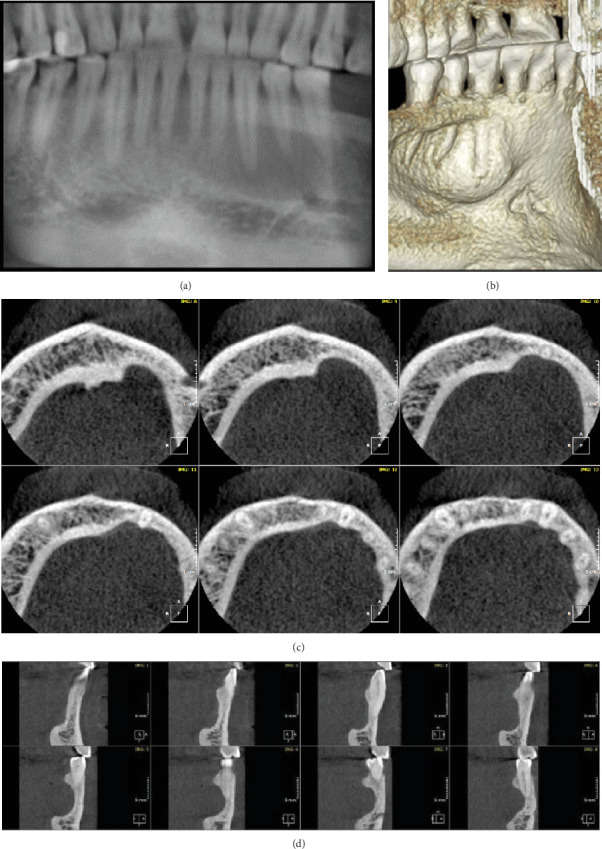
Case 4. CBCT scan with the following views: (a) reconstructed panoramic, (b) 3D rendering reformatting image represents of the extends and area of the bone defect from the inner surface of the mouth, and (c) serial axial cuts representing the curvature and shape of the lingual depression from deep at the bottom to saucering/scalloping. (d) Corrected coronal sectional images show the area of appreciated lingual defect at the left body of the mandible.

A 47-year-old male patient was referred by the oral surgeon for assessment of a radiographic finding in the anterior mandible. CBCT scan, limited view (250 × 250 mm); resolution is 0.2 mm voxel size, presented a significantly large, well-defined, corticated bony defect on the lingual side of the left mandible. The lesion extended from the apical region of Tooth #32 to the distal area of Tooth #35, measuring 5.5 × 18.5 × 6.6 mm (height × width × depth). It runs from the midroot level to 3 mm apical to the root apex of Tooth #33. Radiographically, the defect appeared as a radiolucent area, presenting as saucering of the lingual cortical bone, accompanied by a fenestration and partial root exposure of Teeth #33, #34, and #35. Regarding these radiographic features, the most probable diagnosis was an anterior SBD. Clinical correlation as well as follow-up was recommended in the event of any symptoms. (Figures 4a, 4b, 4c, and 4d).

## 3. Discussion

Four patients with SBDs were evaluated with CBCT. This study illustrates the utility of CBCT in assessing SBDs and highlights their variability, providing a deeper understanding of their radiographic characteristics. In Case 1, we reported a classic posterior mandibular SBD during the implant planning process. This well-defined, corticated radiolucent defect is consistent with the typical dimensions reported by Philipsen et al., who found that 90% of lingual mandibular bone depressions are posterior. Its location, inferior and lingual to the inferior alveolar nerve, corresponds to the most common variant described in the literature [1, 3]. In Cases 2 and 3, the variants were more atypical. Approximately 50 cases have been described in literature involving an anterior mandibular SBD in a 28-year-old female. The smaller size (10 × 5 × 3.5 mm) and associated teeth diversion are unusual features not commonly associated with SBDs. This case underscores the importance of considering SBD in the differential diagnosis of anterior mandibular radiolucencies, even in younger patients and females, who are less commonly affected [2, 6].

Case 3 presented a particularly special scenario with bilateral lingual depressions and additional buccal involvement. This presentation challenges the typical unilateral nature of SBDs that were described in most studies [1, 2, 7]. The etiology of the defect most probably affected the whole lower anterior mandible. According to Philipsen et al., only 0.5% of cases involved buccal depressions, implementing the presence of both buccal and lingual bone loss extremely rare.

Case 4, which manifests as an anterior salivary gland defect, grows our understanding of SBD variability. The lesion's size, along with the adjacent teeth's partial root exposure and associated fenestration, are interesting features. This case is comparable to Katz et al.'s findings, which showed that anterior mandibular SBDs can be challenging to diagnose due to their rarity and potential to mimic various diseases [14].

The radiographic features interpreted in our cases, particularly the well-defined corticated margins and radiolucent appearance, are consistent with those reported in most studies [2, 11]. However, the diversion of the teeth noted in Cases 2, 3, and 4 is not commonly detected with further investigation. Türkoğlu and Orhan reported that anterior SBDs might cause teeth displacement and root diversions, which is similar with our observations in Case 4 [11].

### 3.1. Role of CBCT in Diagnosis of SBDs

Our application of CBCT for diagnostic purposes is consistent with current literature highlighting its significance in the evaluation of SBD [8, 9, 15]. Compared to conventional two-dimensional radiography and MDCT scans, CBCT offers several critical advantages:

1. Three-dimensional imaging: CBCT offers intricate 3D visualizations, facilitating precise analysis of lesion characteristics such as size, shape, and their spatial relationships with adjacent anatomical structures. Case 1 only presented typical radiographic features of SBD and can be diagnosed by panoramic radiographs, where the capability of 3D imaging was significantly advantageous in Cases 2, 3, and 4, where atypical presentations necessitated a more detailed radiographic evaluation.
2. Enhanced diagnostic precision: Conventional radiographic techniques, including panoramic imaging, often fail to accurately represent the true extent of SBDs due to issues like superimposition and distortion. CBCT addresses these shortcomings by providing more reliable assessments. Research by Shimizu et al. indicated that both CT and CBCT imaging yield superior clarity in defining lesion boundaries and enable more reliable differentiation from other conditions, particularly in complex scenarios [15].
3. Evaluation of adjacent structures: The three-dimensional visualization capabilities of CBCT enhance diagnostic confidence by allowing for detailed examination of neighboring teeth and cortical bone. This is particularly relevant in instances of root exposure or fenestration, as illustrated in Case 4. The technology enables a thorough evaluation of how lesions affect nearby anatomical components, including tooth roots and the mandibular canal, which is critical for effective treatment planning.
4. Radiation safety and dose efficiency: Basically, CBCT involves higher radiation than 2D imaging, such as panoramic radiography; its superior diagnostic capabilities justify its use in complex cases, where detailed imaging is necessary. When assessing the benefits against the risks, the greater accuracy of CBCT in locating the entity supports its use for proving the detection of SBDs. This provides the greater radiation exposure associated with CBCT contrasted to the restricted diagnostic potential of panoramic radiographs [16]. On the other hand, CBCT is associated with a significantly reduced radiation dose in comparison to MDCT scans, rendering it a more suitable option for repeated imaging during lesion monitoring or follow-up assessments. This benefit, coupled with its high level of diagnostic accuracy, positions CBCT as an optimal imaging technique for the evaluation of SBDs. It serves as a crucial asset in the precise diagnosis of these lesions, particularly in atypical cases, and in informing appropriate management strategies [13–15]. If we compare the use of CBCT in dental clinics directly with the practice of first obtaining a panoramic radiograph and then referring the patient for MDCT interpretation, employing CBCT from the outset reduces overall radiation exposure while providing comparable diagnostic accuracy to the previously widely used MDCT.

### 3.2. Conservative Management and Follow-Up

Our case series study corroborates the conservative management recommended by the majority of practitioners [3, 4, 12]. Surgical intervention was not an option in any of our cases, further supporting the idea that SBDs are typically benign and stable entities that can be effectively monitored through radiographic imaging. A study conducted by Assaf et al., which analyzed 14,005 panoramic radiographs, revealed a prevalence rate of 0.10% for SBDs, underscoring their infrequency and importance and the critical need for precise diagnosis to prevent unwarranted procedures [12].

## 4. Conclusion

In conclusion, the presented case series illustrates variability in the presentations of SBDs and highlights the requirement for CBCT imaging to develop adequate interpretation and validate a suspected clinical diagnosis. It is important that although SBDs can be expected to follow similar radiographic patterns, clinicians should recall numerous variable possibilities of location, size, and lesion effect on adjacent structures, which are generated by this relatively asymptomatic entity. The variety of SBD manifestations encountered in our study (from the most common posterior to the uncommon anterior and bilateral types) broadens existing knowledge and emphasizes the significance of competent oral radiology examinations in suggested cases. The CBCT examination represents an image modality offering reliable diagnosis without unnecessary recurrence of misinterpretations regardless of challenging anatomical landmarks or dental dislocation-inducing conditions requiring a surgical intervention. Consequently, CBCT-based diagnosis can facilitate avoidance of unnecessary surgeries due to potential presence of suggestive but nonpathognomonic plain radiographic signs.

## Data Availability

The data that support the findings of this study are available on request from the corresponding author. The data are not publicly available due to privacy or ethical restrictions.
